# Efficacy and safety of stem cell therapy for the early-stage osteonecrosis of femoral head: a systematic review and meta-analysis of randomized controlled trials

**DOI:** 10.1186/s13287-020-01956-5

**Published:** 2020-10-19

**Authors:** Lianghao Mao, Pan Jiang, Xuan Lei, Chenlie Ni, Yiming Zhang, Bing Zhang, Qiping Zheng, Dapeng Li

**Affiliations:** 1 Affiliated Hospital of Jiangsu University, Jiefang Road No.438, Zhenjiang, 212001 Jiangsu China; 2 School of Medicine, Jiangsu University, Zhenjiang, Jiangsu China

**Keywords:** Osteonecrosis of femoral head, Stem cells, Collapse of femoral head, Total hip replacement, Progression events, Meta-analysis

## Abstract

**Background:**

Osteonecrosis of femoral head (ONFH) is a seriously degenerative disease with no effective therapies to slow its progression. Several studies have reported short-term efficacy of stem cells on early-stage ONFH. However, its long-term effect was still unclear especially on progression events. This study was performed to evaluate the long-term efficacy and safety of stem cells and analyze its optimal age group and cell number.

**Methods:**

Our review was registered on PROSPERO (http://www.crd.york.ac.uk/PROSPERO), registration number CRD42020136094. Following PRISMA guideline, we searched 8 electronic databases on January 5, 2020, and rigorous random controlled trials (RCTs) utilizing stem cell therapy on early-stage ONFH were included. Quality and bias were analyzed. Pooled analysis was performed to assess difference between various outcomes.

**Results:**

A total of 13 RCTs (619 patients with 855 hips) were included. The application of stem cells significantly delayed collapse of femoral head(*I*^2^, 70%; RR, 0.54; 95% CI, 0.33 to 0.89; *P* < .00001) and total hip replacement (THR) (*I*^2^, 68%; RR, 0.55; 95% CI, 0.34 to 0.90; *P* = .02) in the long term. It effectively decreased the events of collapse of femoral head (≥ 60 months) (*I*^2^, 0%; RR, 0.37; 95% CI, 0.28 to 0.49; *P* < .00001) and THR (> 36 months) (*I*^2^, 0%; RR, 0.32; 95% CI, 0.23 to 0.44; *P* < .00001). There existed a beneficial effect for patients under 40 (Collapse of femoral head: *I*^2^, 56%; RR, 0.41; 95% CI, 0.23 to 0.76; *P* = .004) (THR: *I*^2^, 0%; RR, 0.31; 95% CI, 0.23 to 0.42; *P* < .00001). In addition, quantity of stem cells at 10^8^ magnitude had better effects on disease progression events (*I*^2^, 0%; RR, 0.34; 95%CI, 0.16 to 0.74; *P* = .007). Besides, there were no significant differences on adverse events between the stem cell group and control group (*I*^2^, 0%; RR, 0.82; 95% CI, 0.39 to 1.73; *P* = .60).

**Conclusion:**

Our findings build solid evidence that stem cell therapy could be expected to have a long-term effect on preventing early-stage ONFH patients from progression events, such as collapse of femoral head and total hip replacement. Furthermore, patients under 40 may be an ideal age group and the optimal cell number could be at 10^8^ magnitude for this therapy. Further studies including strict RCTs are required to evaluate a clear effect of stem cells on ideal patient profile and the procedures of implantation.

## Introduction

Osteonecrosis of femoral head (ONFH) is a common orthopedic disease characterized by interruption of blood supply and necrosis of the subchondral bone, subsequently leading to collapse of femoral head [1]. It is usually related to the ischemia of femoral head, increased intraosseous pressure, and metabolism disorders which break the balance between bone absorption and bone remolding. Current operation procedures include core decompression (CD), vascularized bone graft, osteotomy, transplanting tissue engineer materials, and total hip replacement (THR) [2–5]. Unfortunately, there is still no effective therapy that could delay the progression of ONFH [6]. Besides, early intervention before the subchondral fracture would achieve better outcomes. Unfortunately, most patients would usually miss this valuable early period and have to choose THR when diagnosed. Especially for the young, THR has a relatively limited effect due to their higher requirements for activity.

Recently, numerous studies have investigated that stem cells could be a promising therapy for curing bone defects as they can differentiate into specific cells and continue to proliferate to repair damaged tissues. Hernigou et al. [7] first implanted autologous bone marrow stem cells (BMSCs) into necrotic area of femoral head and found that patients had better outcomes if they received larger number of progenitor cells. There are several procedures of various stem cell therapies including combining stem cells with CD, autologous bone graft, platelet-rich plasma, or supporting biomaterial implantation [8–13]. Several studies [12, 14, 15] also confirmed that stem cells could effectively improve early-stage ONFH patients’ symptoms such as pain and hip function in the short term. However, its long-term efficacy and safety remain unclear and controversial particularly on progression events including collapse of femoral head and THR [16, 17]. Besides, due to high heterogeneity among different studies, it is rather difficult for surgeons to determine ideal patients, cell quantity, and methods of implantation for this therapy.

Thus, the main purpose of this study was to systematically evaluate long term efficacy and safety of stem cells by applying rigorous RCTs on early-stage ONFH utilizing stem cells. Progression of ONFH was judged by events of collapse of femoral head, THR, and survival of hips. We also aimed at analyzing the ideal age group and optimal quantity of stem cells therapy based on existing RCT studies.

## Methods

### Protocol and registration

This systematic review and meta-analysis followed the PRISMA (Preferred Reporting Items for Systematic Reviews and Meta-Analyses) guidelines and checklist. This study was preregistered on PROSPERO (https://www.crd.york.ac.uk/PROSPERO/) under number CRD42020136094 before data collection.

### Search strategies and selection criteria

A comprehensive literature search was conducted employing 8 electronic databases (PubMed, Embase, Web of Science, Cochrane Library, ScienceDirect, EBSCO, CINAHL, OVID). The date of publications was restricted up to January 5, 2020, and no language restriction. The following search items and corresponding MESH terms were combined: (Stem cell, Progenitor Cell) AND (Femur Head Necrosis, Aseptic Necrosis of Femur Head, Ischemic Necrosis of Femoral Head). We performed a systematic search including articles, meeting essays, systematic review, reviews, comments, and registered clinical trials.

Two trained investigators independently screened on study titles, abstracts, and full-text manuscripts for eligibility and disagreements were resolved by consensus of a third investigator. The inclusion and exclusion criteria for studies followed the PICOS strategy. The inclusion criteria were following:

*Participants*. Patients diagnosed with early stage of ONFH (Association Research Circulation Osseous stage; ARCO stage, 1 to 3) without any limitation

*Intervention*. Any kind of stem cell therapy

*Comparator*. Any kind of therapy

*Outcomes*. Clinical efficacy and adverse events

*Study*. Randomized controlled trials

Exclusion criteria included studies that did not meet the aforementioned inclusion criteria.

### Data extraction and collection

A data extraction sheet was previously formulated according to the template given by Cochrane Consumers and Communication Review Group. Two independent investigators extracted data and filled into the corresponding sheet independently. A third investigator then verified the accuracy of the synthesized data and disagreements were resolved by consensus. The extracted data were as follows: first author, year of publication, number of patients, age, number of hips and stage of ONFH (ARCO), total cell counts, intervention and comparison, follow-up, adverse events, collapse of femoral head, THR, and survival of hip.

The original authors were contacted to get the unpublished or unclear data. When numeric values were only accessible in graphs, we utilized the software Engauge Digitizer (v 4.1) to extract these data [18]. In studies with duplicate outcomes, data from the original study or study with larger sample size was extracted.

### Quality and bias assessment

The risk of bias and quality of RCTs were assessed by 2 investigators independently, using the Cochrane Collaboration tool. The following factors were assessed in each study: random sequence generation (selection bias), allocation concealment, blinding of participants, outcome assessments, attrition bias (incomplete outcome data), reporting bias (selective reporting), and other biases. The possibility of publication bias was assessed by a funnel plot combined with Egger test using Stata v14.0 software and *P* < .10 indicated significant asymmetry and publication bias.

### Statistical analysis

Meta-analysis was performed with a software, Review Manager (v5.3; Cochrane Collaboration). We used *I*^2^ value to describe the statistical heterogeneity. All reported *P* values are 2-sided and a high value of *I*^2^(> 50%) and *P* < .05 indicate statistically significant heterogeneity among studies for an outcome. A random effect model was carried out when *I*^2^ > 50%, while a fixed effect model was adopted when *I*^2^ < 50%. Sensitivity analyses were employed to judge the impact of individual study on overall estimate and test the stability of results using the leave-one-out method. Furthermore, treatment outcomes were measured and converted into mean differences (MDs) or standard mean differences (SMDs) and 95% confidence intervals (CIs). Subgroup analyses preplanned were employed to evaluate the stability of results on the collapse of femoral head and THR by follow-up time, mean age and numbers of stem cells.

## Results

### Selection of included studies

**Fig. 1:**
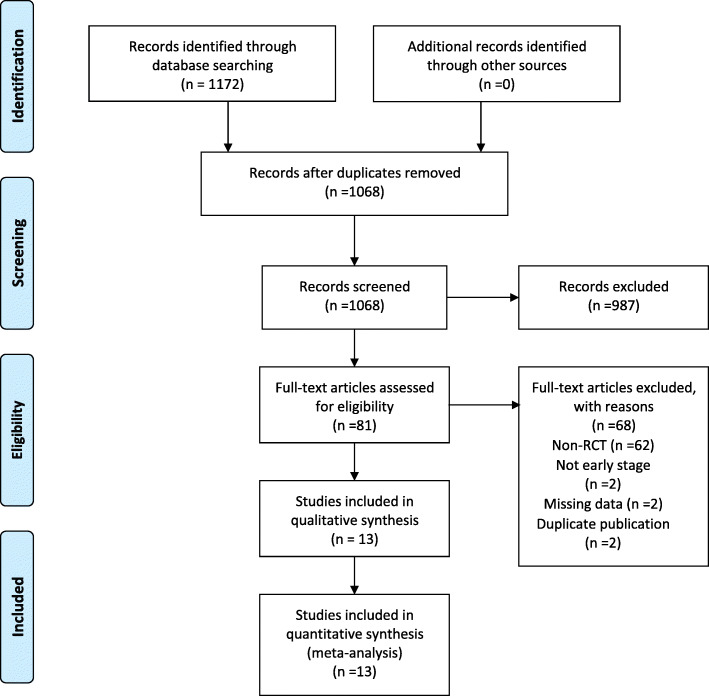
Flow diagram

Initially, a total of 1172 studies were identified through searching multiple databases. 1091 articles were excluded after screening title and abstract because they did not meet the inclusion criteria. Subsequently, 81 studies were assessed for eligibility by reviewing full-text. Of these, 68 studies were excluded for various reasons. Finally, 13 randomized controlled clinical trials were selected for this meta-analysis. The complete selection process is depicted in a flow diagram (Fig. 1).

### Study characteristics

Among these RCTs, 619 patients including 855 hips diagnosed with early-stage ONFH were included. Follow-up time ranged from 2 to 25 years. The range of total cell counts was from 2 × 10^6^ to 3.46 ± 0.36 × 10^9^. All of these studies employed autologous stem cells using the procedure of centrifugation in different kinds of therapies. Twelve studies isolated stem cells from autologous bone marrow [19, 20, 22–31] and one from autologous peripheral blood [32]. Three studies expanded the number of stem cells in vitro [19, 20, 30] and the rest studies chose to inject bone marrow concentration directly [22–29, 31, 32]. Nine studies were performed with CD combined with bone marrow autologous concentrates (BMACs) [23–26, 28–31, 33]. Two studies were performed with mechanical support combined with bone marrow mesenchymal stem cells [19, 27]. One study was performed with porous tantalum rod implantation combined with targeted intra-arterial infusion of peripheral blood stem cells [32]. Another one study was performed with CD combined with bone marrow-derived osteoblastic cells [20]. Detailed characteristics are summarized in Table 1.

**Table 1:** Summary of studies’ characteristics

Author	Patient, *n*	Age, intervention	Age, control	Disease stage	Source of SCs	Isolation of SCs	Preparation of SCs	Expansion of SCs	Intervention, hips	Control, hips	Cell counts	Outcome measure	Follow-up, year
Chang et al. [19]	8	35.7 (19–43)		II/IIIA	Autologous BM	Centrifugation	BMSCs+decalcified bone matrix	DMEM/F12 with 10% autoserum	CD+BMSCs, 8	CD, 8	2 × 10^6^	Collapse of femoral head	2
Gangji et al. [20]	60	50.8 ± 13.2	51.1 ± 10.6	I/II	Autologous BM	Centrifugation	Isolate MSC from BM aspirate	Differentiate and expand osteoblastic cells ex vivo	CD+marrow-derived osteoblastic cells, 30	CD+BMAC, 30	20 × 10^6^	THR	3
Gangji et al. [31]	19	42.2 ± 2.6	45.7 ± 2.8	I/II	Autologous BM	Centrifugation	No	No	CD+BMSCs, 13	CD, 11	92.6 ± 22.4 × 10^7^	Collapse of femoral head, THR, survival of hips	5
Hauzeur [24]	38	48.0 ± 2.8	49.7 ± 3.2	III	Autologous BM	Centrifugation	No	No	CD+BMSCs, 23	CD+saline injection, 23	19.45 ± 3.51 × 10^6^	Collapse of femoral head, THR, survival of hips	2
Hauzeur (2019)	53	50 ± 12	51 ± 10	I/II	Autologous BM	Centrifugation	No	No	CD+BMSCs, 26	CD+osteoblastic cell, 27	9.2 ± 9.5 × 10^6^	Collapse of femoral head, THR	3
Hernigou et al. [23]	125	36 (18–54)		I/II	Autologous BM	Centrifugation	No	No	CD+BMSCs, 125	CD, 125	9.0 ± 2.5 × 10^4^	Collapse of femoral head, THR	25
Ma et al. [27]	39	35.60 ± 8.05	34.78 ± 11.48	I/II/III	Autologous BM	Centrifugation	Bone marrow buffy coat seeded on the cylindrical bone before implantation	No	CD+autologous bone graft+bone marrow buffy coat, 25	CD+autologous bone graft, 24	3 × 10^9^	Collapse of femoral head, THR	2
Mao [34]	55	34.60 (11.50)	36.12 (11.34)	I/II/IIIA	Autologous peripheral blood	Centrifugation	G-CSF at a dosage of 10 μg/kg for 4 days	No	Porous tantalum rod implantation+targeted intra-arterial infusion of PBSCs, 48	Porous tantalum rod implantation, 41	2.47 ± 0.5 × 10^8^	Collapse of femoral head, THR, survival of hips	3
Pepke et al. [25]	24	44.3 ± 3.4	44.5 ± 3.3	II	Autologous BM	Centrifugation	No	No	CD+BMAC, 11	CD, 14	Not mentioned	Survival of hips	2
Rastogi et al. [28]	40	34.67 ± 7.02	33.0 ± 7.71	I/II/III	Autologous BM	Centrifugation	No	No	CD+marrow-derived mononuclear cells, 30	CD+unprocessed bone marrow, 30	1.1 × 10^8^	Collapse of femoral head, THR	2
Ramesh et al. [29]	40	Not mention		I/II	Autologous BM	Centrifugation	No	No	CD+marrow-derived mononuclear cells, 26	CD, 25	5 × 10^8^	Survival of hips	2
Tabatabaee et al. [26]	18	31 ± 11.4	26.8 ± 5.8	I/II/III	Autologous BM	Centrifugation	No	No	CD+BMAC, 14	CD, 14	5 ± 2 × 10^8^	THR	2
Zhao [30]	100	32.7 ± 10.5	33.8 ± 7.70	I/II	Autologous BM	Centrifugation	No	Proliferation in vitro for two weeks	CD+BMMSC, 53	CD, 44	2 × 10^6^	THR	5

*BM* bone marrow, *BMMSC* bone marrow mesenchymal stem cells, *PBSCs* peripheral blood stem cells, *THR* total hip replacement

### Assessment for risk of bias

**Fig. 2:**
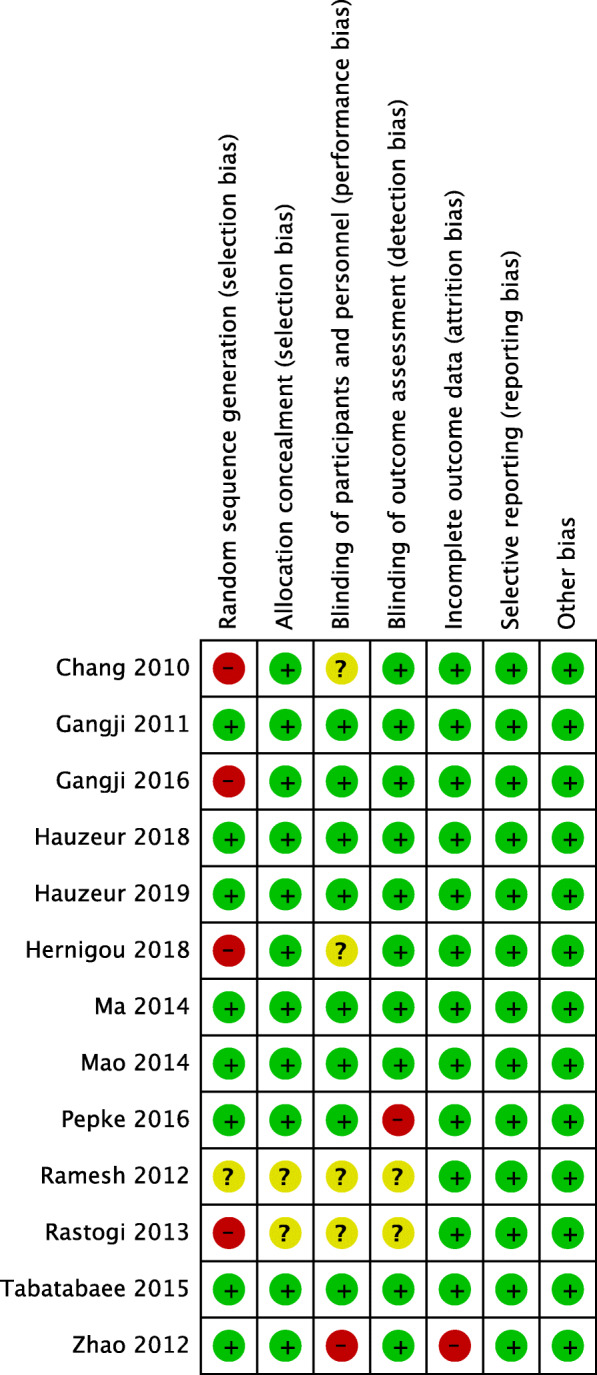
Summary of the risk-of-bias assessment for included studies

Summary of the risk-of-bias assessment for included studies is presented in Fig. 2 Regarding selective reporting, all studies have a relative low risk of bias. For the incomplete outcome data, one study showed high risk of bias [30]. With respect to random sequence generation, 4 studies exhibited a high risk of bias [19, 20, 23, 28] and 1 study had some concerns [29]. In addition, there were 2 studies with some concerns on allocation concealment [28, 29]. For blinding of participants and personnel, 4 studies showed some concerns [19, 23, 28, 29] and 1 study had a high risk of bias [30]. Finally, 2 studies existed some concerns [28, 29] and 1 study showed a high risk of bias for blinding of outcome assessment [25].

### Collapse of femoral head

**Fig. 3:**
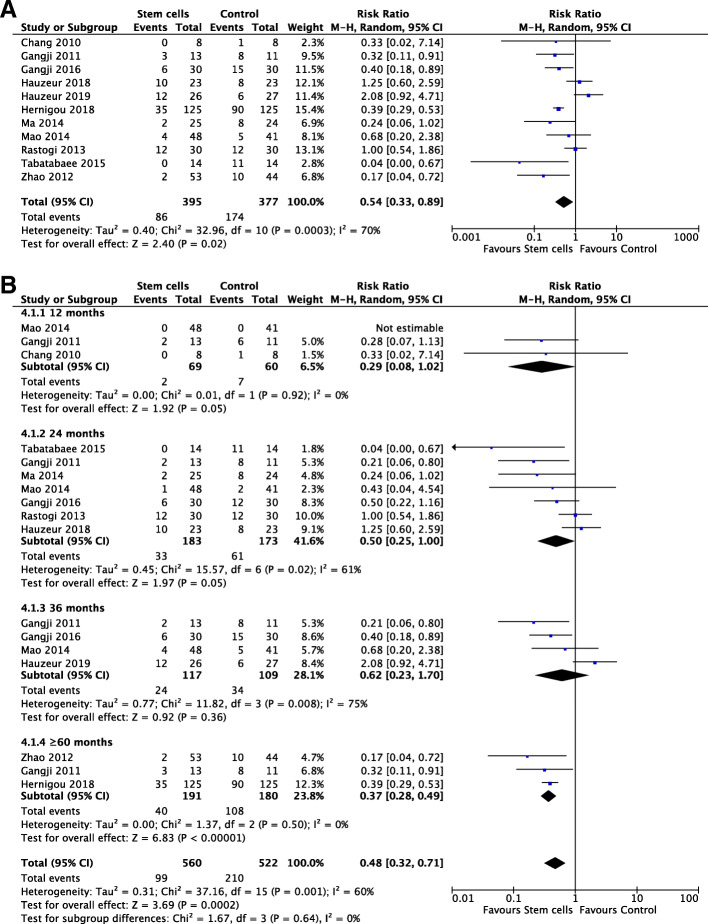
Forest plots in collapse of femoral head. **a** collapse of femoral head at last follow-up. **b** Subgroup analysis on months

Eleven studies [19, 20, 22–24, 26–28, 30–32] reported collapse of femoral head during follow-up including 772 hips (*n* = 395, stem cells; *n* = 377, control). There was a significantly lower events of collapse of femoral head in stem cell group at final follow-up with a high heterogeneity adopting a random effects model (*I*^2^, 70%; RR, 0.54; 95% CI, 0.33 to 0.89; *P* < .00001) (Fig. 3a).

Time of follow-up, age, and number of stem cells were regarded as important sources of heterogeneity. To assess whether the effect of stem cells was linked with time, subgroup analysis was adopted at various time points: 12 months, 24 months, 36 months, and ≥ 60 months after operation. Pooled analysis from 3 studies at more than 60 months demonstrated that stem cell group had significantly lower collapse of femoral head than the control group using a fixed effects model (*I*^2^*,* 0%; RR, 0.37; 95% CI, 0.28 to 0.49; *P* < .00001). However, there were no statistical differences between these two groups on collapse of femoral head at 12 months, 24 months, and 36 months (*P* ≥ .05) (Fig. 3b). Due to high heterogeneity existed in subgroups with follow-up at 24 months and 36 months, we then did a sensitivity analysis and found that after excluding one study by Hauzeur et al. [22], there was a significant difference of pooled effect on the group at 36 months using a random effects model (*I*^2^, 0%; RR, 0.40; 95% CI, 0.22 to 0.72; *P* = .0003).

The subgroup analysis on age illustrated a significant improvement was found in stem cell group for patients under 40 with a random effects model (*I*^2^, 56%; RR, 0.41; 95% CI, 0.23 to 0.76; *P* = .004). Sensitivity analysis was employed to analyze its potential heterogeneity and after excluding one study by Rastogi et al. [28], a significant difference of pooled effect was found with a random effects model (*I*^2^, 5%; RR, 0.36; 95% CI, 0.25 to 0.52; *P* < .00001). Nevertheless, it showed no statistical difference between these two groups for patients ranging from 40 to 50 and more than 50 (*P* ≥ .05) (Supplementary Fig. 1a). Subgroup analysis of stem cell number did not show a significant difference between each group with various magnitude of cells quantity (*P* ≥ .05) although the overall effect was significant using a random effects model (*I*^2^, 70%; RR, 0.54; 95% CI, 0.33 to 0.89; *P* = .02) (Supplementary Fig. 1b). Sensitivity analysis showed that a statistical difference existed in groups with cell number at 10^6^ magnitude using a random effects model (*I*^2^, 0%; RR, 0.38; 95% CI, 0.28 to 0.50; *P* < .00001) after excluding one study by Hauzeur et al. [22]. Besides, the pooled effect of subgroup with cell number at 10^8^ magnitude significantly changed after removing the study by Rastogi et al. [20, 28] utilizing a random effects model (*I*^2^, 26%; RR, 0.0.32; 95% CI, 0.14 to 0.72; *P* = .006).

### Total hip replacement

**Fig. 4:**
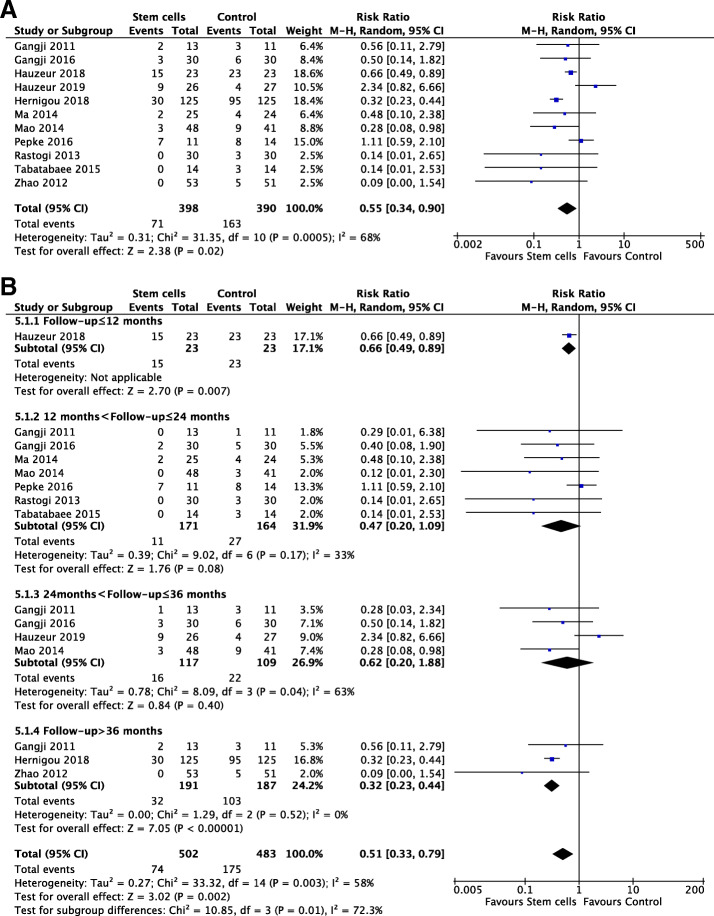
Forest plots in THR. **a** THR at last follow-up. **b** Subgroup analysis on months

Eleven studies [20, 22–28, 30–32] including 788 hips (*n* = 398, stem cells; *n* = 390, control) reported THR. There was a significantly lower THR in stem cells group employing a random effects model (RR, 0.55; 95% CI, 0.34 to 0.90; *P* = .02). The corresponding *I*^2^ (68%) indicated a substantial heterogeneity (Fig. 4a).

Subgroup analysis was employed to investigate the potential source of heterogeneity. Pooled analysis showed that stem cell group had significantly lower THR during follow-up more than 36 months utilizing a fixed effects model (*I*^2^, 0%; RR, 0.32; 95% CI, 0.23 to 0.44; *P* < .00001). It also raised a significant difference between two groups at a follow-up less than 12 months (RR, 0.66; 95% CI, 0.49 to 0.89; *P* = .007). However, there were no significant difference between groups at a follow-up from 12 to 24 months and 24 to 36 months, respectively (*P* > .05) (Fig. 4b). Due to medium heterogeneity existed the subgroups with a follow-up from 24 months to 36 months, we then did a sensitivity analysis and noticed that after excluding one study by Hauzeur et al. [22]; there was a statistical difference of pooled effect with no heterogeneity using a random effects model (RR, 0.36; 95% CI, 0.16 to 0.81; *P* = .01).

Similar to the analysis of collapse of femoral head, subgroup analysis of THR on age showed that there only existed a significant difference among the patients groups less than 40 using a random effects model (*I*^2^, 0%; RR, 0.31; 95% CI, 0.23 to 0.42; *P* < .00001) (Supplementary Fig. 2a), which had no statistical difference in the other two groups ranging from 40 to 50 and more than 50 (*P* ≥ .05). In addition, subgroup analysis of stem cells number illustrated a significant difference on the rate of THR among the groups with 10^8^ magnitude of cells quantity utilizing a random effects model (*I*^2^, 0%; RR, 0.34; 95% CI, 0.16 to 0.74; *P* = .007) (Supplementary Fig. 2) and no statistical difference between groups at 10^6^ or 10^7^ magnitude. Sensitivity analysis to investigate high heterogeneity in the subgroup with 10^6^ cell number revealed that there was a statistical difference after excluding study by Hauzeur et al. [22] using a random effects model (*I*^2^, 0%; RR, 0.31; 95% CI, 0.22 to 0.43; *P* < .00001).

### Survival of hip

Five studies [24, 25, 29, 31, 32] including 235 hips (*n* = 121, stem cells; *n* = 114, control) reported the survival of hip. Three studies [29, 31, 32] found that there was a significant difference of hips in the interval from operation to THR between the stem cells group and control group, illustrating that stem cell therapy may have an advantage on hip survival (*P* < .05). However, 2 studies [24, 25] including patients in ARCO stage II and III respectively reported that there were no statistical differences in the hip survival time between CD combined with bone marrow autologous concentrates (BMACs) and CD alone (*P* > .05).

### Safety analysis

Twelve studies [19, 20, 22–25, 27–32] reported an incidence of adverse events after operation including pain, fever, nausea, hematoma in the trochanter region, infection including positive bacterial culture, and porous tantalum rod displaced. This meta-analysis found that there were no significant differences on adverse events between the stem cell group and control group with no heterogeneity using a fixed effects model (*I*^2^, 0%; RR, 0.82; 95% CI, 0.39 to 1.73; *P* = .60) (Supplementary Fig. 3).

### Publication bias

Visual inspection of the funnel plot for collapse of femoral head showed symmetry in studies reporting collapse of femoral head and THR (Supplementary Fig. 4a, Supplementary Fig. 4b). In addition, Egger’s test for the asymmetry of the funnel plot did not suggest significant evidence of publication bias (ICC, 0.099; *P* = .951 for collapse of femoral head) (ICC, 0.863; *P* = .883 for THR).

## Discussion

The most important finding of this meta-analysis was that the application of stem cells could significantly postpone the disease progression and increase the survival of hip in the long term. Our results first indicated that stem cell therapy could effectively decrease the events of collapse of femoral head (≥ 60 months) and THR (> 36 months). Furthermore, we initially concluded that stem cell therapy would be more beneficial for patients under 40 comparing other generations. In addition, this study showed that the optimal quantity of stem cells would probably be at 10^8^ magnitude which would have fewer progression events.

Several previous studies [12, 13, 21, 35] focused on cell therapies have found that there was a clinical improvement of symptoms on patients with ONFH including pain and function scores. However, the efficacy of stem cells in the long term is not clear and remains controversial especially on the progression events [9, 11, 12, 36, 37]. Until now, there was no strict meta-analysis of RCTs to investigate the effect of this therapy on ONFH in the long term. Besides, due to the high heterogeneity between different studies, it is difficult to determine the standard procedure of this therapy and optimal patients [38–40]. In this case, it is urgent to investigate the efficacy and safety of stem cell therapy on ONFH by performing a meta-analysis utilizing rigorous RCTs with high level of evidence.

This meta-analysis showed that therapy with stem cells for early-stage ONFH could significantly have better outcomes on preventing collapse of femoral head and delaying the time for THR in the long term contributing to a better survival. Safety analysis also showed that there were fewer adverse events such as pain, fever, and nausea by adopting this therapy [20, 22–24], which was also supported by several other studies [13, 41, 42]. Besides, compared with control groups, we found that a significant difference was existed on the collapse of femoral head during follow-up of more than 60 months and THR head more than 36 months. When using sensitivity analysis to analyze heterogeneity among these studies, we found that the study by Hauzeur et al. [22] may be the source since its control group was treated by CD plus osteoblastic cells which was quite different from other studies. Considering the instability of some results by sensitivity analysis, we cannot draw strong interferences that whether there is better improvement of stem cell therapy in the short term. According to a study by Houdek et al. [12], CD associated with BMACs plus platelet-rich plasma could significantly improve symptoms and more than 90% of hips in this group did not collapse over 2 years. Similarly, Nally et al. [11] reported that CD with BMSCs had THR at during the follow-up of 5.5 years. Therefore, injection stem cells are a promising and safe therapy to protect patients on early-stage ONFH from further disease progression.

In addition, we investigated the proper age of patients for stem cell therapy and found that patients under 40 would gain fewer progression events including collapse of femoral head and THR. Furthermore, our subgroup analysis showed that the reasonable quantity of stem cells may be at 10^8^ magnitude in terms of better long-term benefits. Nevertheless, our sensitivity analysis found that high sensitivity existed in 2 studies by Hauzeur et al. [22] and Rastogi et al. [28]. After analyzing these studies, we found that different from other trials, Rastogi et al. [28] only reported the quantity of mononuclear cells but did not give specific number of stem cells which may cause some heterogeneity as we treated them as the total number. The heterogeneity in the study by Hauzeur et al. [22] was explained before. On account of the instability in these results judged by sensitivity analysis, it is rather difficult for us to conclude that whether 10^6^ was a logical magnitude for stem cells in order to get better survival. Why do people under 40 have much better outcomes in the long run than older generations? We hold the view that it may be due to the aging-associated decline of potential pluripotency of stem cells. Previous studies found that older age would have higher serum levels of RANKL and lower level of IGF-1 which could deteriorate the bone regeneration and osteogenic differentiation medicated by stem cells [43, 44]. Besides, the microenvironment of bone marrow could be quite different among different ages which also affect the potential and biodistribution of stem cells. Several researchers investigated that older patients usually have complicated microenvironment changes characterized with dysregulation of metabolism and immune system influenced by various epigenetic factors and signaling networks [45–48]. In addition, different from our results, a previously [6] published guideline on ONFH suggested that effectiveness of stem cells are limited on preserving joint and the revision was relative high due to young patients. We believed that this difference may due to the heterogeneity arising from limited studies and procedures but basing on our analysis of strict RCTs there was a better effect on preventing progression events for patients under 40.

However, the mechanisms behind stem cell therapy remained unclear and may be partly explained by the theory of biological characteristics [49, 50]. Since stem cells have the capabilities of self-renew and proliferation, when injected into necrotic femoral head, they could differentiate into osteoblast, chondrocyte, and other tissues to repair dead bones [51]. Apart from this, stem cells could also secrete multiple biological factors such as various growth factors, cytokines, and exosomes to promote angiogenesis and rebuild blood supply, which would inversely decrease the intraosseous pressure and prevent the progression of ONFH [52–54]. Kang et al. [55] found that bone marrow mononuclear cells with calcium phosphates could enhance the expression of VEGF and promotes osteogenesis stimulating new trabecular bone remolding. Gagala et al. [56] investigated that combing BMSCs with osteochondral allograft would provide structural support and promote articular and bone regeneration. In addition, another study [52] found that BMSCs exposed to hypoxia environment could increase the level of genes concerning bones metabolism, including alkaline phosphatase, Type I collagen, and osteocalcin, stimulating repairing activities in ONFH. Consequently, stem cell therapy may be a promising method to improve the progression of ONFH and more studies are required to investigate its therapeutic effects and mechanisms.

For this meta-analysis, we firstly investigated the proper age of patients and optimal quantity of stem cells to magnify their therapeutic effect on ONFH by combing strict RCTs. Besides, although the evidence of improvement on disease progression was relatively limited in the short term, the outcomes on collapse of femoral head and THR were significantly decreased in the long term. The limitations of this study should be there existed a heterogeneity on treatment procedures between these RCTs, since most RCTs were performed with CD combined with BMSCs but 3 studies [19, 27, 32] were performed with mechanical support and other forms of stem cells. However, given that the control group also adopted the same mechanical support, differences in various clinical outcomes were regarded as an effect of stem cells. Second, the contents of injection cells were complicated as bone marrow stem cells were different from BMSCs or PBSCs. After standard centrifuging and sorting, bone marrow stem cells were then expanded in vitro and finally injected into patients’ body. Moreover, ONFH has diverse etiologies characterized with different pathologies; however, we could not evaluate the effect of stem cells on ONFH patients with different etiology, stage or sex due to the shortage of such RCTs data.

## Conclusion

Our findings build solid evidence that stem cell therapy could be expected to have a long-term effect on preventing early-stage ONFH patients from progression events, such as collapse of femoral head and total hip replacement. Furthermore, patients under 40 may be an ideal age group and the optimal cell number could be at 10^8^ magnitude for this therapy. Further studies including strict RCTs are required to evaluate a clear effect of stem cells on ideal patient profile and the procedures of implantation.

## Supplementary information

**Additional file 1 : Supplementary Fig. 1.** Forest plots in collapse of femoral head. **a** Subgroup analysis on age. **b** Subgroup analysis on cell number. **Supplementary Fig. 2.** Forest plots in THR. **a** Subgroup analysis on age. **b** Subgroup analysis on cell number. **Supplementary Fig. 3.** Forest plots in adverse events. **Supplementary Fig. 4.** Funnel plots in progression events. **a** collapse of femoral head. **b** THR.

## Data Availability

All supporting data are included in the article and its supplementary files.

## Abbreviations

ONFH: Osteonecrosis of femoral head
RCTs: Random controlled trials
CD: Core decompression
THR: Total hip replacement
BM: Bone marrow
BMSCs: Bone marrow stem cells
PRISMA: Preferred reporting items for systematic reviews and meta-analyses
ARCO: Association Research Circulation Osseous stage
MDs: Mean differences
SMDs: Standard mean differences
CIs: Confidence intervals
BMACs: Bone marrow autologous concentrates
BMMSC: Bone marrow mesenchymal stem cells
PBSCs: Peripheral blood stem cells
