# Supplementary material for: Efficacy and safety of stem cell therapy for the early-stage osteonecrosis of femoral head: a systematic review and meta-analysis of randomized controlled trials
Source: Stem Cell Res Ther. 2020 Oct 19;11:445. doi: 10.1186/s13287-020-01956-5 (PMC7574494; doi:10.1186/s13287-020-01956-5)
Supplement: Supplementary file 1 — Additional file 1 : Supplementary Fig. 1. Forest plots in collapse of femoral head. a Subgroup analysis on age. b Subgroup analysis on cell number. Supplementary Fig. 2. Forest plots in THR. a Subgroup analysis on age. b Subgroup analysis on cell number. Supplementary Fig. 3. Forest plots in adverse events. Supplementary Fig. 4. Funnel plots in progression events. a collapse of femoral head. b THR. [file 13287_2020_1956_MOESM1_ESM.doc]

**Supplementary Fig. 1** Forest plots in collapse of femoral head. **a** Subgroup analysis on age. **b** Subgroup analysis on cell number.

**Supplementary Fig. 2** Forest plots in THR. **a** Subgroup analysis on age. **b** Subgroup analysis on cell number.


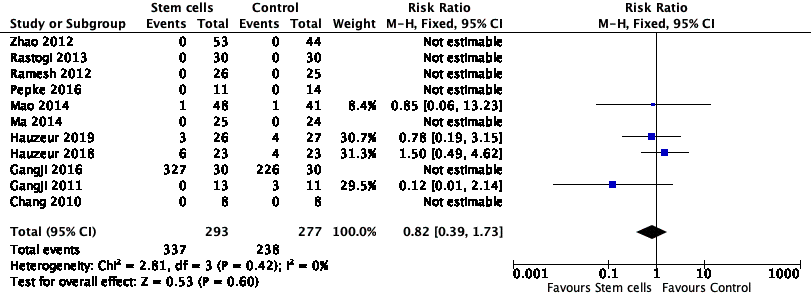


**Supplementary Fig. 3** Forest plots in adverse events.


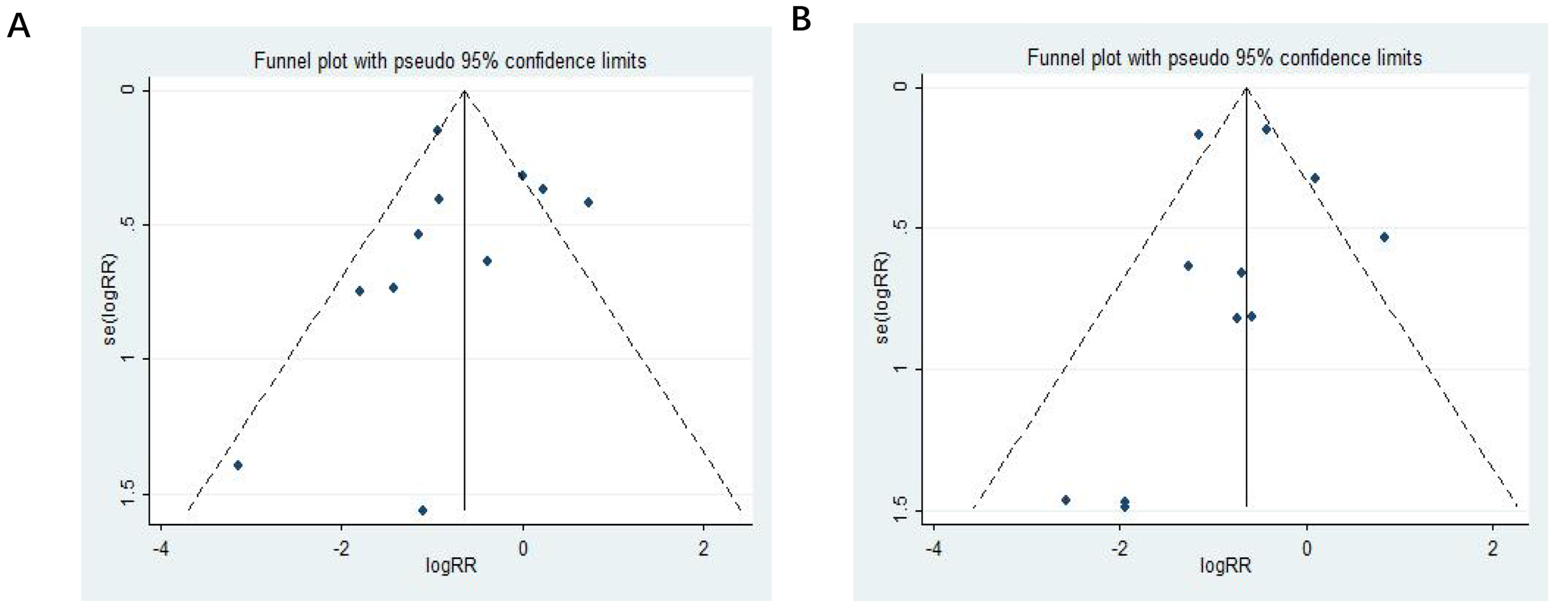


**Supplementary Fig. 4** Funnel plots in progression events. **a** collapse of femoral head. **b** THR.
